# JunB Mediates Basal- and TGFβ1-Induced Smooth Muscle Cell Contractility

**DOI:** 10.1371/journal.pone.0053430

**Published:** 2013-01-04

**Authors:** Aruna Ramachandran, Samudra S. Gangopadhyay, Ramaswamy Krishnan, Sandeep A. Ranpura, Kavitha Rajendran, Sumati Ram-Mohan, Michelle Mulone, Edward M. Gong, Rosalyn M. Adam

**Affiliations:** 1 Urological Diseases Research Center, Boston Children’s Hospital, Boston, Massachusetts, United States of America; 2 Department of Surgery, Harvard Medical School, Boston, Massachusetts, United States of America; 3 Center for Vascular Biology Research, Beth Israel Deaconess Medical Center, Boston, Massachusetts, United States of America; 4 Department of Medicine, Harvard Medical School, Boston, Massachusetts, United States of America; William Harvey Research Institute, Barts and The London School of Medicine and Dentistry, Queen Mary University of London, United Kingdom

## Abstract

Smooth muscle contraction is a dynamic process driven by acto-myosin interactions that are controlled by multiple regulatory proteins. Our studies have shown that members of the AP-1 transcription factor family control discrete behaviors of smooth muscle cells (SMC) such as growth, migration and fibrosis. However, the role of AP-1 in regulation of smooth muscle contractility is incompletely understood. In this study we show that the AP-1 family member JunB regulates contractility in visceral SMC by altering actin polymerization and myosin light chain phosphorylation. JunB levels are robustly upregulated downstream of transforming growth factor beta-1 (TGFβ1), a known inducer of SMC contractility. RNAi-mediated silencing of JunB in primary human bladder SMC (pBSMC) inhibited cell contractility under both basal and TGFβ1-stimulated conditions, as determined using gel contraction and traction force microscopy assays. JunB knockdown did not alter expression of the contractile proteins α-SMA, calponin or SM22α. However, JunB silencing decreased levels of Rho kinase (ROCK) and myosin light chain (MLC20). Moreover, JunB silencing attenuated phosphorylation of the MLC20 regulatory phosphatase subunit MYPT1 and the actin severing protein cofilin. Consistent with these changes, cells in which JunB was knocked down showed a reduction in the F:G actin ratio in response to TGFβ1. Together these findings demonstrate a novel function for JunB in regulating visceral smooth muscle cell contractility through effects on both myosin and the actin cytoskeleton.

## Introduction

The function of hollow organs such as the urinary bladder is dependent on appropriate contractility of smooth muscle (SM). In response to pathologic stimuli, such as mechanical stress, or altered innervation, smooth muscle cells (SMC) undergo phenotypic changes that result in loss of differentiation markers, cellular hypertrophy, increased production of extracellular matrix proteins and eventual loss of contractile function [1]. Although the consequences of such tissue remodeling are evident by the prevalence of diseases associated with aberrant SM function, the molecular mechanisms that regulate SM phenotype in hollow organs other than the vasculature are still incompletely understood.

The AP-1 transcriptional complex has been implicated in pathologic changes in smooth muscle exposed to injury. Previous observations from our group implicated discrete AP-1 species as mediators of PDGF-stimulated SMC migration [2] and stretch-induced expression of fibrogenic proteins in visceral SMC [3]. In addition, transforming growth factor-beta 1 (TGFβ1) is a ubiquitous cytokine and a key regulator of smooth muscle differentiation in diverse organ systems (reviewed in [4]). Gene deletion studies in mice revealed that loss of one allele of TGFβ1 led to decreased expression of canonical SM contractile proteins [5]. Alternatively, SM-specific ablation of the type II TGFβ receptor in smooth muscle cells (SMC) during development led to compromised differentiation of aortic SM and embryonic lethality [6]. Consistent with a role for TGFβ1 in contractile protein expression regulation, elevation of TGFβ1 in hollow organs has been linked to alterations in muscle contractility through direct effects on SM marker expression. TGFβ1 has also been shown to upregulate expression of profibrotic proteins that ultimately alter tissue compliance [7]–[9]. In addition, TGFβ1 can affect cell contractility by altering components of the actin cytoskeleton. Interestingly, exposure of cells in culture to TGFβ1 increases stress fiber formation, which in turn can feed forward to regulate SM marker expression in SMC in a RhoA-dependent manner [10], suggesting functional interaction between the actin cytoskeleton and the smooth muscle contractile apparatus.

AP-1 is known to mediate the effects of TGFβ1 on target gene expression in a variety of cell types [11]. TGFβ1-stimulated increases in AP-1 activity underlie expression of SM contractile proteins, including α-SMA and SM22α [12], [13]. Furthermore pharmacologic inhibition of AP-1 with T-5224, a small molecule inhibitor, can abrogate TGFβ1-induced fibrosis [14]. However, the role of AP-1 in regulating visceral SMC contractility has not been explored. In this study, we investigated the functional significance of AP-1 in regulating contractility in SMC. These studies reveal a novel role for JunB as an effector of both basal and TGFβ1-stimulated contractility.

## Materials and Methods

### Ethics Statement

These studies were performed in strict accordance with the recommendations in the Guide for the Care and Use of Laboratory Animals of the National Institutes of Health. The protocol was approved by the Animal Care and Use Committee of Boston Children’s Hospital (11-03-1925R). All surgeries were performed under isoflurane anesthesia and every effort was made to minimize suffering.

### Cell Culture

Primary human bladder smooth muscle cells (pBSMC) were cultured in DMEM supplemented with 10% fetal bovine serum (FBS, Valley Biomedical, Winchester, VA), 2 mM L-glutamine, penicillin (100 U/ml), and streptomycin (100 µg/ml)(all from Invitrogen, Carlsbad, CA) at 37°C in a humidified atmosphere of 95% air-5% CO_2_. All experiments were performed on cells between passages 3 and 6.

### Gel Contraction Assay

pBSMC at a density of 150,000 cells/ml were suspended in a solution of neutralized rat tail type I collagen to a final concentration of 1.2 mg/ml [15](BD Biosciences, San Jose, CA), seeded in 24-well plates and placed at 37°C for 1 h to polymerize. Following overnight equilibration in medium containing 0.5% FBS, cells were treated with 2.5 ng/ml TGFβ1 in medium containing 0.5% FBS for 24 h. In selected experiments, cells were co-incubated with TGFβ1 and pharmacologic inhibitors of PI3K (LY294002), Akt (triciribine), MEK (PD98059), p38 SAPK (SB202190), JNK (SP600125) and Rho kinase (Y27632)(all at 10 µM). Following treatment, gels were released and the extent of gel contraction was monitored over time on an imaging workstation. In each case, the control condition was set to 100 percent and all other values were calculated relative to that.

### Traction Force Microscopy

To measure pBSMC cell contraction, we implemented a recently described technique called monolayer traction microscopy [16]. Briefly, polyacrylamide gel substrates were prepared by mixing acrylamide, bis-acrylamide (Bio-Rad, Hercules, CA), and 0.5 µm diameter yellow fluorescent beads (Invitrogen, Eugene, OR) in ultrapure water. The mixture was then added to the center of pretreated 20 mm diameter glass-bottomed wells of 6-well plates (In Vitro Scientific, Sunnyvale, CA). After polymerization, gel surfaces were activated by adding 200 µl of 1 mM sulfosuccinimidyl-6-(4-azido-2-nitrophenylamino)hexanoate solution (Pierce, Rockford, IL) and exposed to UV light for 6 min. The gels were then washed and ligated with collagen type I. The final gel stiffness was 4 kPa. Following nucleofection with non-targeting or JunB siRNA oligos, 100,000 pBSMC were added to gels and incubated overnight in DMEM supplemented with 10% fetal bovine serum. Following serum depletion for 24 h, cells were treated without or with TGFβ1 (2.5 ng/ml) for a further 24 h, at which point contractile forces were measured. For each well, we recorded a spatial map of fluorescent beads that were embedded within the gel substrate directly underneath the pBSMC cells, as described [16]. Following detachment of cells from substrates using 0.05% trypsin, we obtained a second spatial map of the same fluorescent beads. By comparing the two maps, monolayer displacement fields could be calculated. From the monolayer displacement field and with knowledge of substrate stiffness, we computed the monolayer traction field as described previously [16]. For each condition, we pooled traction values over all regions of each monolayer and across all monolayers. From this pooled set, we computed the median value and the standard error for each treatment condition.

### Transcription Factor ELISA

To examine activation of multiple AP-1 subunits concurrently, we employed a commercially available AP-1 family Transcription Factor ELISA (Active Motif, Carlsbad, CA). Nuclear extracts were prepared from cells treated with 2.5 ng/ml TGFβ1 (R&D Systems, Minneapolis, MN) or vehicle for 8 or 24 h essentially as described [2]. Protein was quantified using the BioRad *DC* protein assay (BioRad Laboratories, Hercules, CA). Three to 5 µg nuclear extracts were used in the TF ELISA, which was performed according to the manufacturer’s instructions.

### Immunofluorescence Staining

pBSMC were seeded on sterile glass cover slips in complete medium at a density of 1×10^5^ cells per well in 6-well plates. Twenty-four h later, cells were subjected to serum depletion for a further 24 h in medium containing 0.5% FBS. Cells were treated without or with 2.5 ng/ml TGFβ1 for 24 h. Cells were fixed in 4% paraformaldehyde for 15 min at RT, rinsed with PBS 4 times (3 min each) and blocked with PBS/1% BSA/0.1% Triton X-100 for 1 h at RT. Primary antibody to JunB (C37F9, Cell Signaling Technology, Danvers, MA) at 1∶200 dilution in PBS/1% BSA/0.1% Triton X-100 was added and cells incubated overnight at 4°C in a moist chamber. Cells were rinsed 4 times with PBS (5 min each), and Cy3-conjugated secondary anti-rabbit antibody (1∶500 dilution in PBS/1% BSA/0.1% Triton X-100) was added for 1 h at RT, protected from light. Cells were rinsed 4 times with PBS (5 min each) and mounted with Vectamount containing DAPI prior to visualization of sections using a Zeiss Axioplan-2 fluorescence microscope (Carl Zeiss MicroImaging, Inc. Thornwood, NY). For IIF staining of tissues, sections were deparaffinized in xylene, rehydrated through graded ethanols, equilibrated in PBS for 10 min and blocked for 1 h at RT in PBS/1% serum. JunB antibody (1∶100 in PBS/1% serum/1% BSA/3% Triton X-100) was incubated with the sections overnight at 4°C in a moist chamber. Subsequent processing of the sections was carried out as described above.

### Knockdown of JunB by siRNA

To target expression of JunB in pBSMC, ∼2.4×10^6^ cells were nucleofected with 100 pmol of either non-targeting (control) or JunB-specific siRNA oligonucleotides (Dharmacon, Lafayette, CO) using program A-033 on a Nucleofector IIN (Amaxa, Inc., Gaithersburg, MD). Approximately 1.8×10^5^ cells from each nucleofection reaction were seeded in plastic plates, in collagen gels for gel contraction evaluation or on collagen-coated polyacrylamide gels for traction force microscopy. Extent of silencing was determined by semi-quantitative RT-PCR using gene-specific primers as described [2], or by immunoblot analysis.

### Immunoblot Analysis

Following TGFβ1 treatment, cells were lysed with 1X lysis buffer (20 mM Tris-Cl (pH 7.5), 150 mM NaCl, 1% Triton X-100, 0.5% SDS, 1 mM EDTA, 1 mM EGTA, 2.5 mM NaPPi, 1 mM β-glycerophosphate, 1 mM NaF, 1 mM Na_3_VO_4_, 1 µg/ml leupeptin). To reduce viscosity, lysates were passed through a 30G needle six times, followed by centrifugation at 16,000×g for 10 min at 4°C and quantification using the MicroBCA assay (Pierce Chemical Co., Rockford, IL). Samples were resolved by SDS-PAGE, electrotransferred to nitrocellulose membranes and blocked with 10% dried milk in PBS/0.1% Tween 20 before overnight incubation with primary antibodies. Membranes were washed 3×15 min in PBS/0.1% Tween 20, incubated with species-specific secondary antibodies for 1 h at RT and signals were visualized by enhanced chemiluminescence (SuperSignal West Pico reagent, Pierce Chemical Co) and exposure of membranes to film. Quantitation of protein levels was carried out using data from at least three independent experiments (representative blots are shown in inserts). Protein levels were normalized to their respective GAPDH levels and expressed as fold change relative to cells transfected with control siRNA and not subjected to TGFβ1 treatment, unless stated otherwise.

### Assessment of F:G-actin Ratio

Alterations in the F:G-actin ratio in pBSMC treated with control or JunB-targeted siRNAs were determined using the G-actin/F-actin in vivo assay kit (Cytoskeleton, Inc., Denver, CO), essentially according to the manufacturer’s instructions. Briefly, cells were lysed in a buffer that solubilizes G-actin but renders F-actin insoluble. Following high-speed centrifugation (100,000×g at 37°C for 1 h), F-actin was recovered in the pellet, whereas G-actin remained in the supernatant. The pellet was resuspended in ice-cold distilled water to the same volume as the supernatant and incubated on ice with intermittent pipetting for 1 h in the presence of 10 µM cytochalasin D in order to dissociate F-actin. Laemmli buffer was added to equal volumes of the supernatant and the resuspended pellet, which were then resolved by SDS-PAGE, electrotransferred to nitrocellulose and probed with anti-actin antibody.

### Rodent Bladder Distension Models

An ex vivo model of bladder stretch injury was used as previously described [17]. Briefly, 6–8 wk-old female rats were anesthetized with isoflurane inhalation. The bladder was catheterized and distended to 40 cm water pressure using a gravity manometer with serum-free DMEM. A low midline incision was made to expose the bladder. The bladder neck was isolated and tightened with a 4–0 silk suture. The catheter was removed and the bladder excised. The excised bladder was placed in serum-free DMEM and maintained in culture at 37°C in a humidified 5% CO_2_/95% air atmosphere incubator. As a control, a non-distended bladder was harvested and incubated in parallel with the stretch bladders as a control. Two bladders were employed for each time point (control and stretch-injured). At the end of the incubation period, specimens were decompressed, fixed in 10% neutral buffered formalin at room temperature for 48 h, rinsed with PBS, dehydrated in ethanol and embedded in paraffin. Sections of 8 µm thickness were obtained with microtome and mounted on glass slides. To determine the impact of bladder distension in an intact animal, we also employed an acute bladder outlet obstruction model, essentially as described [18]. Briefly, 6-week old female CD-1 mice under isoflurane anesthesia were subjected to laparotomy to expose the bladder. The proximal urethra was ligated with 6-0 nylon suture, and the mice were recovered following closure of the abdominal wall. Bladder distension was achieved by urine production by the animal over a 24 h period, after which organs were harvested.

### Statistical Analysis

Where appropriate, comparisons between experimental groups were performed using Student’s t-test. P values are indicated in figure legends.

## Results

### TGFβ1 Induces Contractility in Smooth Muscle Cells

**Figure 1:**
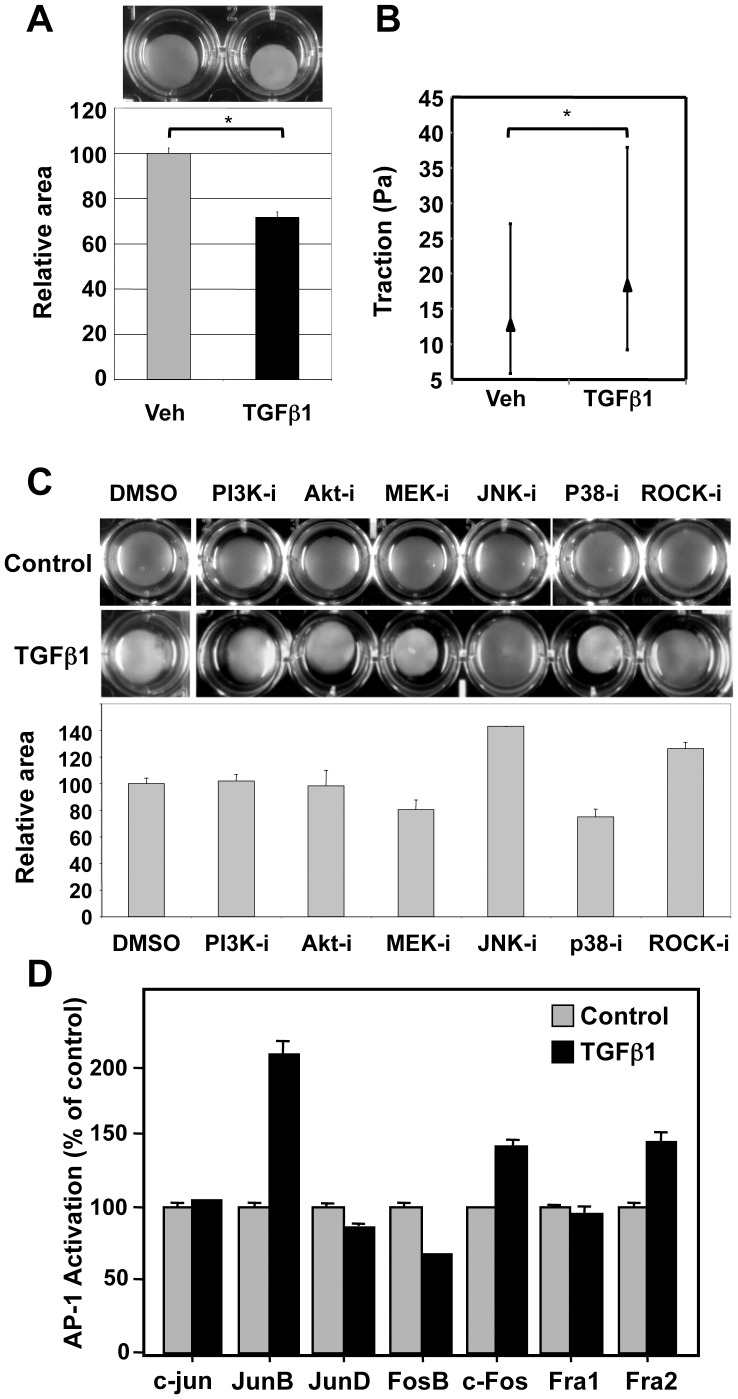
TGFβ1induces contractility in bladder smooth muscle cells (BSMC). (**A**) Human bladder smooth muscle cells were seeded in collagen gels and treated for 24 h with vehicle (Veh) or 2.5 ng/ml TGFβ1, after which the gels were released from the sides of the well and the resulting decrease in surface area monitored microscopically (top) and quantified (bottom). *p<0.05, t-test. The area of the gel under control conditions is set to 100%. (**B**) Whisker plot of results from traction force microscopy of BSMC showing an increase in cell traction forces exerted with TGFβ1 treatment. The contractile response, measured quantitatively as enhanced traction (see Methods) was statistically significant (*p<0.05, Kruskal-Wallis test). The median value of traction and the interquartile range for both groups is shown. (**C**) BSMC were treated for 30 min with inhibitors targeting the PI3-kinase/Akt (PI3K-i, Akt-i) mitogen-activated protein kinases (MEK-i, p38-i, JNK-i) or Rho-kinase (ROCK-i), followed by treatment with vehicle (Control, upper panel of wells) or 2.5 ng/ml TGFβ1 (lower panel) for 24 h and were monitored for changes in gel contractility. Inhibition of signaling via the JNK and ROCK axes abrogated TGFβ1-induced gel contraction. Quantification of changes in gel surface area for the various inhibitors under conditions of TGFβ1 treatment is indicated. (**D**) A transcription factor ELISA was carried out to assess differences in DNA-binding activities of members of the AP-1 family of transcription factors, using nuclear extracts prepared from BSMC treated with 2.5 ng/ml TGFβ1 for 24 h, or control cells. Fold changes are expressed relative to control which is set to 100%.

TGFβ1 is known to regulate growth, differentiation and contractility of SMC from different organ sites [4]. Previous data from our group identified TGFβ1 as a potent growth inhibitor for smooth muscle cells isolated from different regions of the urinary tract [19]. Here, we determined the impact of TGFβ1 on the contractile phenotype of primary human bladder smooth muscle cells (pBSMC). First, we evaluated contractility using a gel contraction assay, as described previously [15]. TGFβ1 treatment led to increased contractility of pBSMC (Figure 1A), with gel surface area reduced by ∼30% in response to growth factor treatment (p<0.05). Similar changes in cellular traction forces were observed using traction force microscopy (Figure 1B).

TGFβ1 is known to signal through a number of parallel kinase cascades, including the PI3K/Akt, MAPK and Rho kinase pathways [20]. To assess which of these may mediate the effects of TGFβ1 on contractility, the gel contraction assay was performed in the presence of pharmacologic inhibitors of PI3K (LY294002), Akt (triciribine), MEK (PD98059), JNK (SP600125), p38 (SB202190) and ROCK (Y27632). As expected, the ROCK inhibitor, Y27632, a known promoter of SMC relaxation, reversed the TGFβ1-stimulated reduction in gel area (Figure 1C, lower panel, lane 7). Of the other agents tested, only the JNK inhibitor SP600125 was effective in inhibiting TGFβ1-induced pBSMC contractility (Figure 1C, lower panel, lane 5). Cells treated with inhibitors in the absence of TGFβ1 displayed minimal contractility (Figure 1C, upper panel). Similarly, only the JNK and ROCK inhibitors prevented TGFβ1-induced cell contractility in the multipotent progenitor cell line 10T1/2 (data not shown). To investigate potential mechanisms that may underlie the effect of TGFβ1 on pBSMC contractility, we employed a quantitative transcription factor ELISA to assess AP-1 activation. Members of the AP-1 transcriptional complex are known targets of JNK, and have been reported to mediate the effects of TGFβ1 in other cell types [11]–[14]. We screened seven AP-1 subunits for their ability to bind to a consensus AP-1 motif, and observed a robust and selective increase in DNA-binding activity of JunB in BSMC following TGFβ1 treatment (Figure 1D). A similar pattern of selective activation of JunB by TGFβ1 was observed in 10T1/2 cells (data not shown).

### TGFβ1 Induces JunB Expression and Activity in pBSMC

**Figure 2:**
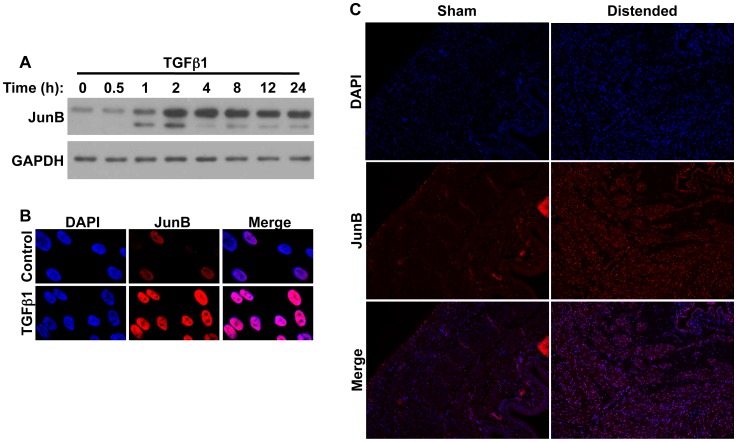
JunB levels are increased in BSMC in response to TGFβ1, and in an ex vivo model of rodent bladder distension. (**A**) BSMC were treated with TGFβ1 for the indicated times and assessed for JunB levels by immunoblotting. GAPDH is included as a loading control. (**B**) Immunofluorescence analysis of BSMC showing increased JunB nuclear localization upon TGFβ1 treatment for 24 h. (**C**) Sections from rat bladders distended ex vivo for 8 h (injured) were stained sequentially with anti-JunB and Cy3-conjugated species-specific secondary antibody. Increased nuclear fluorescent signal for both proteins was evident in the detrusor smooth muscle of stretch-injured specimens, but not of non-distended (control) bladders.

Next, we verified the effect of TGFβ1 on JunB levels in pBSMC by immunoblotting (Figure 2A), and observed a time-dependent increase in JunB level. In independent cultures of SMC, JunB levels peaked from 2–8 h after treatment with TGFβ1. Although levels declined after 8 h, appreciable amounts of JunB were still evident at 24 h. JunB nuclear expression levels were increased in TGFβ1-treated pBSMC, as observed by indirect immunofluorescence (Figure 2B). We went on to investigate expression of JunB in an ex vivo model of bladder injury. Immunofluorescence analysis revealed an increase in JunB expression in the detrusor smooth muscle of rat bladders distended ex vivo for 8 h, compared to sham-operated controls (Figure 2C). A similar increase in JunB in bladder smooth muscle was also observed in a mouse model of acute bladder outlet obstruction (data not shown).

### JunB Knockdown in pBSMC Attenuates Contractility

**Figure 3:**
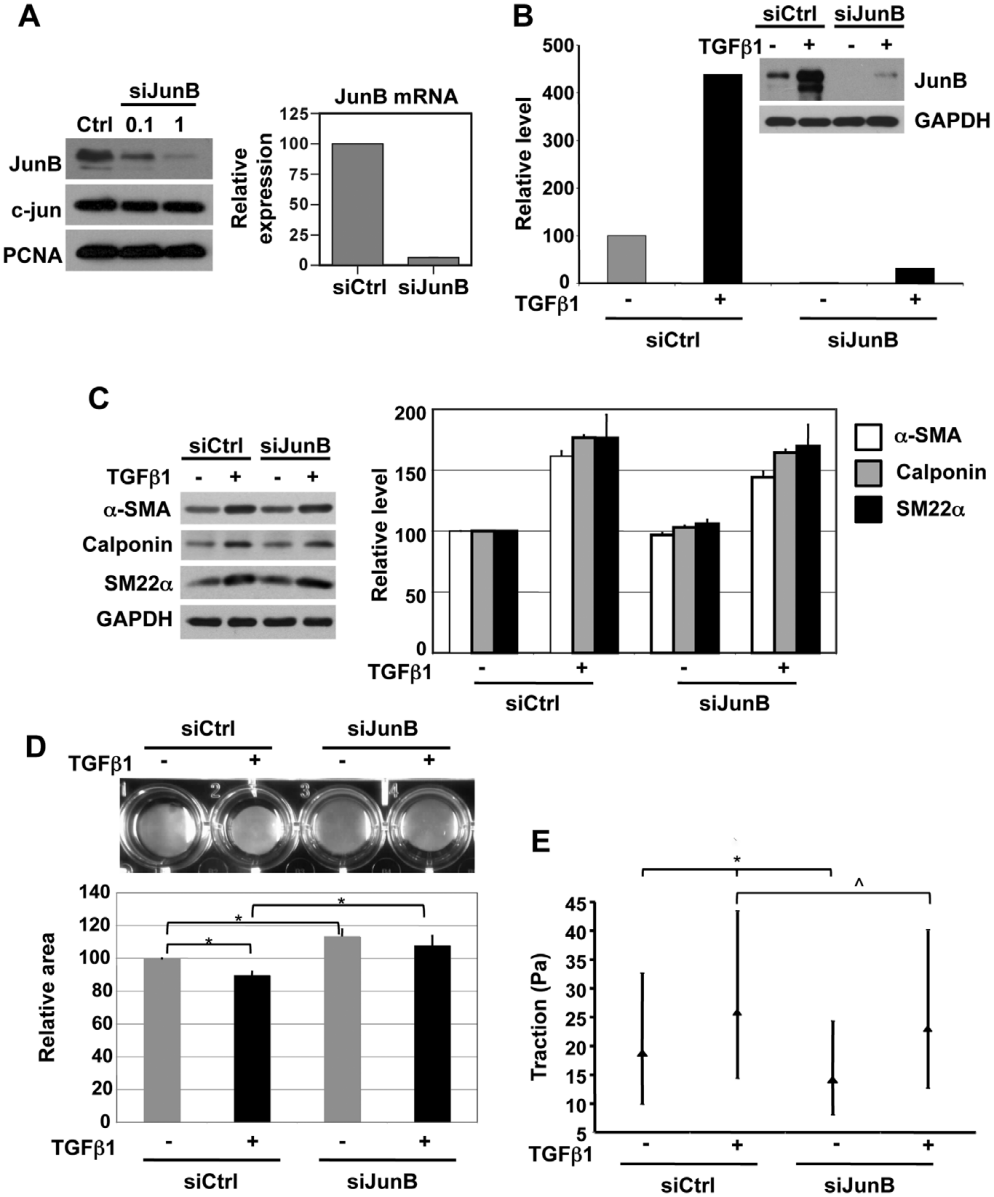
JunB silencing attenuates TGFβ1-induced changes in cell contractility and cytoskeletal tension, but not induction of markers of smooth muscle differentiation. (**A**) BSMC were nucleofected with non-targeting control siRNA or with siRNA against JunB (0.1 µM and 1 µM) and assessed for JunB protein by immunoblotting (left panel, top). Effective knockdown of JunB was observed, with no change in c-Jun levels, demonstrating specificity of the siRNA used. Proliferating cell nuclear antigen (PCNA) expression was used as a loading control. 1 µM JunB siRNA reduced the levels of JunB mRNA by >80%, relative to non-targeting control siRNA, as assessed by semi-quantitative real-time PCR (right panel) (**B**) Reduction in JunB protein levels by siRNA in BSMC under basal and TGFβ1-stimulated conditions, demonstrated by immunoblotting. JunB levels were normalized to their respective GAPDH levels and expressed as percentage change relative to cells transfected with control siRNA and not subjected to TGFβ1 treatment. A representative immunoblot and its corresponding quantitation are shown. (**C**) TGFβ1-mediated induction of α-smooth muscle actin (α-SMA) calponin and SM22α, markers of smooth muscle differentiation, was unaffected by silencing of JunB, as shown by immunoblotting (left). Quantification of immunoblots is shown in the graph (right). Gel contraction assays (**D**) revealed that JunB knockdown significantly reduced both basal and TGFβ1-induced changes in cellular contractility. *p<0.05, t-test (**E**) Inhibition of JunB inhibits basal and TGFβ1-induced contraction. This inhibition of contraction, measured quantitatively as a reduction of traction (see Methods) was statistically significant (*p<0.05, comparing siCtrl+ TGFβ1 or siJunB-TGFβ1 with siCtrl-TGFβ1; ∧p<0.05 comparing siCtrl+ TGFβ1 with siJunB+ TGFβ1 Kruskal-Wallis test). The median value of traction and the interquartile range across all tested groups is shown.

To further test the hypothesis that JunB was a regulator of SMC phenotype, we employed RNA interference to knock down JunB expression and determined the effect on pBSMC cell contractility. Following nucleofection of SMC with JunB siRNA duplexes we observed effective knockdown of JunB at the protein and mRNA level (Figure 3A). Importantly, while a 10-fold increase in the concentration of JunB siRNA led to a dose-dependent decrease in JunB protein level, there was no change in levels of the closely-related AP-1 subunit c-Jun. This implies minimal off-target effects of JunB siRNA. Consistent with the data in Figures 1 & 2, TGFβ1 treatment of pBSMC nucleofected with siCtrl oligos induced JunB expression ∼3-5-fold (Figure 3B, lanes 1, 2). This induction was significantly attenuated under conditions of JunB knockdown (Figure 3B, lanes 3, 4).

JunB silencing did not alter levels of mRNA (not shown) or protein (Figure 3C) for α-SMA, calponin or SM22α inpBSMC in the absence or presence of TGFβ1 treatment. In contrast, however, JunB silencing inhibited both basal and TGFβ1-stimulated contractility of pBSMC as determined in both gel contraction (Figure 3D) and traction force microscopy (Figure 3E) experiments. These findings suggest that, whereas JunB is dispensable for expression of SM contractile proteins, it plays a significant role in regulation of cytoskeletal tension.

### JunB Regulates Contractility through Effects on Both MLC and Actin

**Figure 4:**
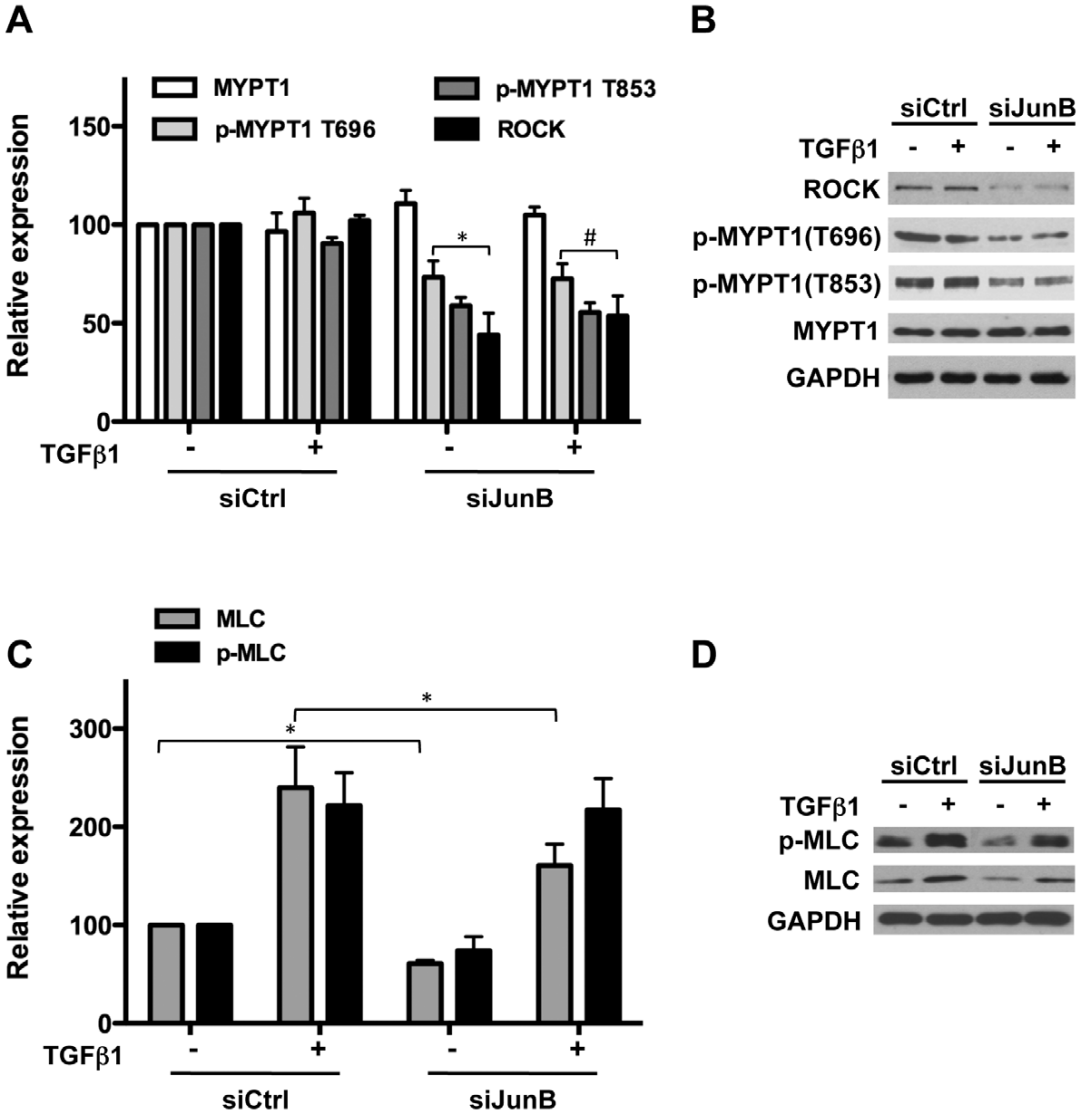
JunB regulates proteins involved in acto-myosin interactions. (**A**) JunB silencing in BSMC significantly reduces basal and TGFβ1-stimulated levels of Rho-kinase (ROCK1) and phosphorylation of myosin phosphatase target subunit 1 (MYPT1) at two activating Thr residues without affecting total protein levels. Representative immunoblots are shown in (**B**). (**C**) Total and phosphorylated myosin regulatory light chain (MLC20) levels are reduced upon JunB knockdown. *p<0.05. Representative immunoblots are shown in (**D**)**.**

Cytoskeletal tension is controlled by diverse elements including actin filaments, actomyosin interactions and microtubules, several of which are downstream of the RhoA-ROCK axis. Initial observations in the gel contraction assay demonstrated reversal of TGFβ1-induced contractility in cells pre-treated with the ROCK inhibitor Y-27632 (Figure 1C). Consistent with a role for ROCK in mediating JunB-dependent effects on pBSMC contractility, we observed a marked decrease in ROCK1 protein levels following JunB knockdown (Figure 4A). Phosphorylation of the ROCK target MYPT1 at Thr696 and Thr853 was also decreased in JunB-silenced pBSMC, with no change in total MYPT1 protein levels (Figure 4A, B). We observed a modest reduction in total and phospho-MLC20 protein levels with JunB knockdown under basal and TGFβ1-treated conditions (Figure 4C, D).

**Figure 5:**
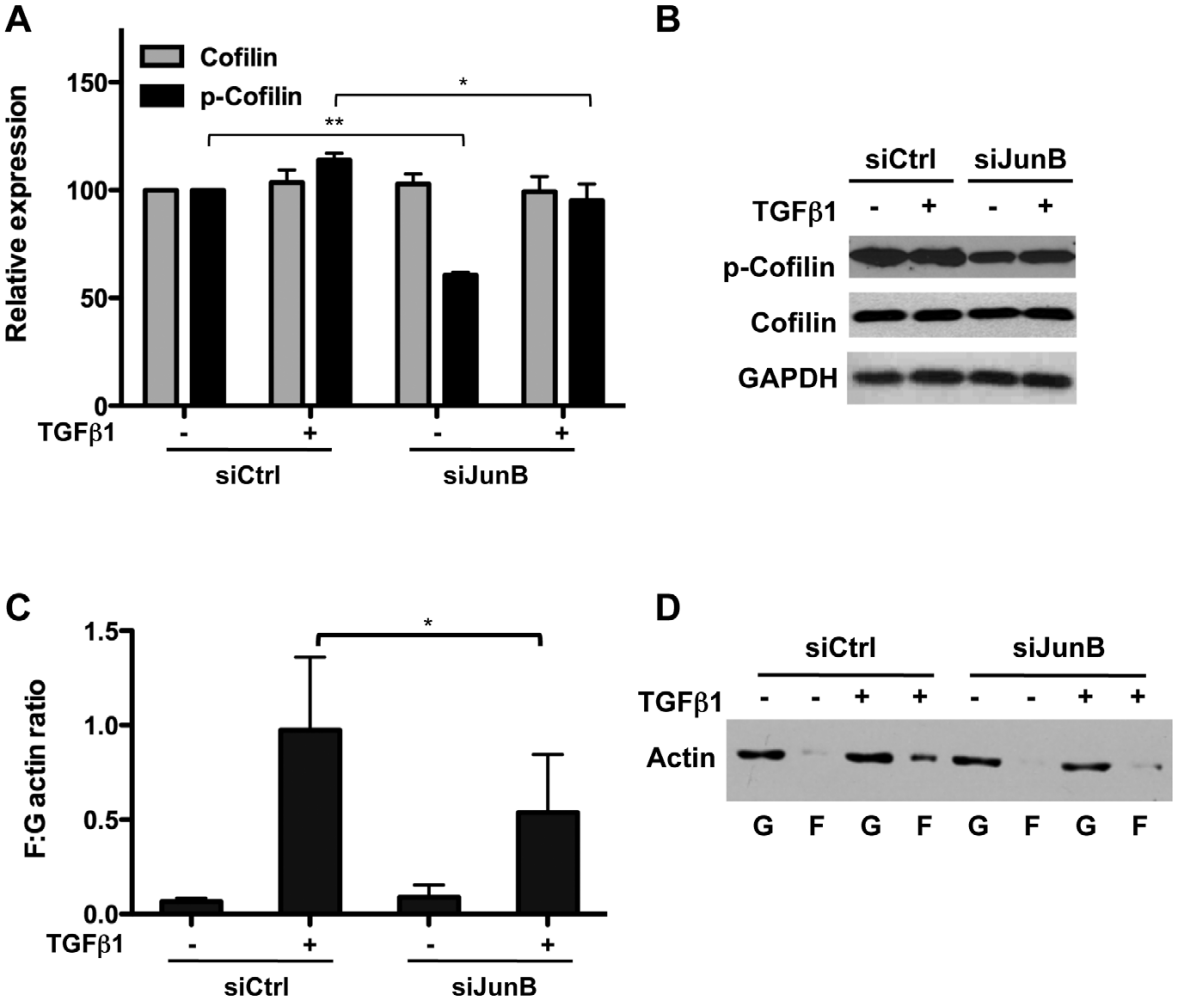
JunB regulates actin polymerization. (**A**) JunB silencing in BSMC reduces phospho-cofilin levels under basal and TGFβ1-stimulated conditions, without affecting total cofilin levels. *p<0.05; **p<0.005. Representative immunoblots are indicated in (**B**). (**C**) Filamentous (F) and globular (G) actin fractions were purified as indicated in Methods, from pBSMC under vehicle or TGFβ1-treated conditions, following treatment with non-targeting or JunB siRNA. The relative levels of F- and G-actin were subsequently assessed by immunoblotting. Quantification of immunoblot signals from three independent experiments is shown. *p<0.05. Representative immunoblots are indicated in (**D**).

Next, we investigated the impact of JunB silencing on the actin cytoskeleton. In agreement with the observed reduction in ROCK levels, JunB knockdown led to decreased phosphorylation of the actin depolymerization factor cofilin, without affecting total cofilin levels (Figure 5A, B) under both basal and TGFβ1-stimulated conditions. Phosphorylation of cofilin inactivates its actin-severing activity, such that reduced phosphorylation would be expected to increase actin depolymerization. Consistent with this, we observed a significant reduction in the F:G actin ratio indicating an increase in depolymerized actin following JunB silencing in pBSMC (Figure 5C, D). These findings are consistent with the decreased ability of JunB-silenced pBSMC to (a) promote contraction of collagen gels (Figure 3D) and (b) exert tension on a deformable substrate, as assessed by traction force microscopy (Figure 3E). Taken together, our results demonstrate a requirement for JunB in regulating actomyosin-mediated contractility in pBSMC.

## Discussion

**Figure 6:**
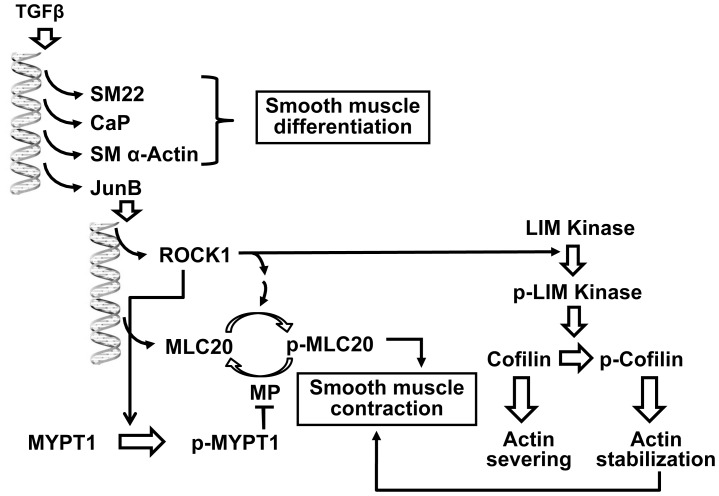
A model depicting the role of JunB in regulation of smooth muscle contractility in response to TGFβ1 signaling. TGFβ1 induces the expression of JunB as well as other markers of smooth muscle differentiation e.g. α-SMA, calponin and SM22α Additionally, TGFβ1 also promotes smooth muscle contraction via ROCK1-mediated regulation of actin polymerization and acto-myosin crossbridge cycling. JunB mediates this process by promoting the phosphorylation of cofilin, leading to stabilization of filamentous actin and also by regulating the phosphorylation and absolute levels of MLC20, the regulatory light chain of myosin, and its inhibitory phosphatase, MYPT1. Thus, activation of JunB is critical for the changes in contractility and generation of cytoskeletal tension observed upon the TGFβ1-stimulation of smooth muscle cells.

In this study, we provide evidence to support a role for JunB as a novel regulator of contractility in visceral SMC. In particular, we demonstrated that siRNA-mediated knockdown of JunB attenuated contractility and cellular traction forces under basal conditions, and in response to a known procontractile agonist, i.e. TGFβ1. Among AP-1 family members JunB emerged as the dominant effector of TGFβ1 in pBSMC. We also showed that JunB elicited its effects on contractility by regulating ROCK levels, MYPT1 phosphorylation, cofilin phosphorylation, which impacted both myosin and actin arms of the contractile apparatus in pBSMC (Figure 6).

TGFβ1-induced contractility in smooth muscle has been reported previously [21]–[23] and JunB mRNA levels are increased by TGFβ1 in various cell types [24]–[28]. However, the extent to which JunB activity contributes to (a) basal levels of contractility and (b) TGFβ1-induced contractility, in visceral SMC has not been described. Smooth muscle contraction is initiated by the phosphorylation of regulatory myosin light chain, MLC20, an event that can be reversed by myosin phosphatase-mediated dephosphorylation. ROCK can phosphorylate both MLC20 and myosin phosphatase. As a result, it can promote cross-bridge cycling either directly via MLC20 phosphorylation or indirectly by phosphorylating the myosin phosphatase targeting subunit MYPT1.

In a recent study, conditional ablation of JunB in vivo using a Col1α2-driven Cre recombinase was found to downregulate expression of *Myl9*, the gene encoding MLC20 in vascular SMC [29]. In contrast, our findings showed that silencing of JunB in pBSMC (i.e. visceral SMC) did not alter *Myl9* mRNA levels, suggesting that JunB-mediated transcriptional regulation of Myl9 may differ in SMC from discrete origins. Knockdown of JunB did lead to a partial reduction in MLC20 protein level under basal conditions that could not be fully rescued following TGFβ1 treatment. In the case of ROCK1, protein levels were sensitive to JunB knockdown independent of TGFβ1 treatment, suggesting a potential role for JunB in regulating ROCK gene expression. Transcriptional regulation of the ROCK promoter has not been investigated in detail. It would be interesting in future studies to explore the role of JunB in this regard. In agreement with the decrease in ROCK levels, phosphorylation of MYPT1 was attenuated in JunB-silenced cells irrespective of TGFβ1 treatment. Although the dependence of ROCK1 expression on JunB is clear, the reasons for the lack of induction of ROCK1 by TGFβ1 are unclear, but may reflect the kinetics employed in these studies or the requirement for additional signals downstream of TGFβ1 stimulation that may compensate for absence of JunB.

In addition to effects of JunB silencing on myosin regulation, a key feature of our study was the identification of JunB as a novel regulator of the actin cytoskeleton in pBSMC. The contribution of actin polymerization to smooth muscle contractility is increasingly appreciated [30]. The dynamics of actin cytoskeletal remodeling are regulated primarily by actin depolymerization proteins, that themselves are targets of kinases such as ROCK. Cofilin is a major regulator of actin depolymerization, activity of which is regulated by phosphorylation. Phosphorylation of cofilin on Ser 3 inhibits its actin-severing activity, shifting the equilibrium of the cellular actin pool from monomeric G to filamentous F-actin [31]. JunB silencing decreased cofilin phosphorylation, consistent with increased cofilin activity, reduced F-actin levels and a corresponding decrease in cell contractility.

A recent report demonstrates that expression of JunB itself is controlled by the dynamics of actin polymerization in the cell [32]. JunB is a direct target of serum response factor (SRF), a ubiquitous transcription factor involved in smooth muscle proliferation, differentiation and contractility (reviewed in [33]), along with megakaryocytic acute leukemia (MAL), an SRF coactivator. MAL binds to and is sequestered in the cytoplasm by actin monomers, preventing it from translocating to the nucleus and activating expression of a subset of SRF-target genes, such as JunB [32], [34]–[36]. In contrast, expression of c-Fos, another AP-1 family member that is a transcriptional target of SRF, is unaffected by actin polymerization levels, demonstrating that SRF alone is sufficient for activation of select target genes, independent of MAL [32]. Notably, expression of both JunB and Fos was required to induce differentiation; neither one alone was sufficient. This is a compelling example illustrating the differential regulation of members of the AP-1 transcription factor family in response to a given stimulus and their subsequent downstream effects.

JunB has also recently been linked to epithelial-mesenchymal transition (EMT) and profibrotic changes induced by TGFβ signaling, in murine mammary epithelial cells [28]. In contrast to our observations in pBSMC, Gervasi and colleagues showed that markers of differentiation in response to TGFβ stimulation were significantly reduced upon JunB silencing, as were levels of tropomyosin (Tpm1), which is required for TGFβ-mediated stress fiber formation. However, in that study no changes in cellular traction forces or contractility under conditions of JunB knockdown were described [28].

Alterations in bladder smooth muscle contractility underlie various conditions afflicting the lower urinary tract, including overactive bladder secondary to neurologic or inflammatory insults, diabetic cystopathy and lower urinary tract symptoms associated with obstruction [1]. Our findings demonstrating JunB as a major TGFβ1 effector, suggests that JunB-mediated alterations in contractility are likely to contribute to the pathologic bladder contractility that occurs following spinal cord injury, a condition in which TGFβ1 is known to be upregulated [8]. Furthermore, transcripts for TGFβ1 and TGFβ1-sensitive genes are upregulated in bladder SMC under conditions of elevated bladder pressure and mechanical stimulation [3], [37]. This is in accordance with our observation of rapid and robust induction of JunB expression and nuclear localization in the detrusor smooth muscle following acute bladder outlet obstruction and wall distension in rodent models of bladder injury. Taken together, these findings suggest that JunB is important for maintenance of basal contractile function in pBSMC and that the TGFβ1-JunB axis is likely to contribute to abnormal smooth muscle contractility associated with lower urinary tract dysfunction. The functional significance and therapeutic relevance of JunB in muscle contractility in vivo will be explored in future studies.
